# Supplementary material for: From sequence to enzyme mechanism using multi-label machine learning
Source: BMC Bioinformatics. 2014 May 19;15:150. doi: 10.1186/1471-2105-15-150 (PMC4229970; doi:10.1186/1471-2105-15-150)
Supplement: Additional file 2 — Java code of ml2db. Additional file ml2db_code.tar.gz contains the Java source code to run the multi-label machine learning experiments and save the results to database. The code’s Javadoc is included. [file 1471-2105-15-150-S2.zip › additional file 2/ml2db/ml2db/doc/main/ml2db/mulan/classifier/lazy/BRkNNFewInstances.html]

BRkNNFewInstances


JavaScript is disabled on your browser.


- Overview
- Package
- Class
- Use
- Tree
- Deprecated
- Index
- Help

- Prev Class
- Next Class

- Frames
- No Frames

- All Classes

- Summary:
- Nested |
- Field |
- Constr |
- Method

- Detail:
- Field |
- Constr |
- Method


main.ml2db.mulan.classifier.lazy

## Class BRkNNFewInstances

- java.lang.Object
- - mulan.classifier.MultiLabelLearnerBase
  - - mulan.classifier.lazy.MultiLabelKNN
    - - main.ml2db.mulan.classifier.lazy.BRkNNVisible
      - - main.ml2db.mulan.classifier.lazy.BRkNNFewInstances

- All Implemented Interfaces:
  :   java.io.Serializable, mulan.classifier.MultiLabelLearner, weka.core.TechnicalInformationHandler

  ---

    

  ```
  public class BRkNNFewInstances
  extends BRkNNVisible
  ```

  An implementation of Mulan BRkNN for Active Learning that allows predictions
  confidence to raise above 50% even when the instances in the training set are
  fewer than (labels - 1).

  Version:
  :   20 Apr 2012

  Author:
  :   Luna De Ferrari ldf(at)st-andrews.ac.uk or
      luna.deferrari@st-andrews.ac.uk

  See Also:
  :   Serialized Form

- - ### Nested Class Summary

    - ### Nested classes/interfaces inherited from class main.ml2db.mulan.classifier.lazy.BRkNNVisible

      `BRkNNVisible.ExtensionType`
  - ### Field Summary

    - ### Fields inherited from class mulan.classifier.lazy.MultiLabelKNN

      `WEIGHT_INVERSE, WEIGHT_NONE, WEIGHT_SIMILARITY`
  - ### Constructor Summary

    Constructors

    Constructor and Description

    `BRkNNFewInstances(int numOfNeighbors)`
  - ### Method Summary

    - ### Methods inherited from class main.ml2db.mulan.classifier.lazy.BRkNNVisible

      `getFile, getTechnicalInformation, globalInfo, printNeighbours, setCvMaxK, setkSelectionViaCV`
    - ### Methods inherited from class mulan.classifier.lazy.MultiLabelKNN

      `isUpdatable, setDfunc, setDistanceWeighting`
    - ### Methods inherited from class mulan.classifier.MultiLabelLearnerBase

      `build, getDebug, makeCopy, makePrediction, setDebug`
    - ### Methods inherited from class java.lang.Object

      `equals, getClass, hashCode, notify, notifyAll, toString, wait, wait, wait`

- - ### Constructor Detail


    - #### BRkNNFewInstances

      ```
      public BRkNNFewInstances(int numOfNeighbors)
      ```

      Parameters:
      :   `numOfNeighbors` -


- Overview
- Package
- Class
- Use
- Tree
- Deprecated
- Index
- Help

- Prev Class
- Next Class

- Frames
- No Frames

- All Classes

- Summary:
- Nested |
- Field |
- Constr |
- Method

- Detail:
- Field |
- Constr |
- Method
